# Phosphate depletion in insulin-insensitive skeletal muscle drives AMPD activation and sarcopenia in chronic kidney disease

**DOI:** 10.1016/j.isci.2023.106355

**Published:** 2023-03-10

**Authors:** Ana Andres-Hernando, Christina Cicerchi, Gabriela E. Garcia, David J. Orlicky, Peter Stenvinkel, Richard J. Johnson, Miguel A. Lanaspa

**Affiliations:** 1 Division of Renal Diseases and Hypertension, University of Colorado Anschutz Medical Campus, Aurora, CO, USA; 2 Division of Endocrinology, Metabolism and Diabetes, University of Colorado Anschutz Medical Campus, Aurora, CO, USA; 3 Division of Nephrology, Rocky Mountain VA Medical Center, Aurora, CO, USA; 4 Department of Pathology, University of Colorado Anschutz Medical Campus, Aurora, CO, USA; 5 Department of Renal Medicine M99, Karolinska University Hospital, SE-141 86Stockholm, Sweden

**Keywords:** Biological sciences, Physiology, Pathophysiology, Cell biology

## Abstract

Sarcopenia is a common and devastating condition in patients with chronic kidney disease (CKD). Here, we provide evidence that the kidney-muscle crosstalk in sarcopenia is mediated by reduced insulin sensitivity and the activation of the muscle-specific isoform of AMP deaminase, AMPD1. By using a high protein-based CKD model of sarcopenia in mice and differentiated human myotubes, we show that urea reduces insulin-dependent glucose and phosphate uptake by the skeletal muscle, thus contributing to the hyperphosphatemia observed in CKD whereas depleting intramuscular phosphate needed to restore energy and inhibit AMPD1. Hyperactivated AMPD1, in turn, aggravates the low energy state in the muscle by removing free adenosine monophosphate (AMP) and producing proinflammatory factors and uric acid which contribute to the progression of kidney disease. Our data provide molecular and metabolic evidence supporting the use of strategies aimed to improve insulin sensitivity and to block AMPD1 to prevent sarcopenia in subjects with CKD.

## Highlights

• Uremic sarcopenia is mediated by low muscle insulin sensitivity and phosphate uptake
• Phosphate balance in plasma and muscle is dysregulated in CKD-mediated sarcopenia
• Blockade of muscle AMP deamination improves renal dysfunction and muscle atrophy

## Graphical abstract

**Figure:**
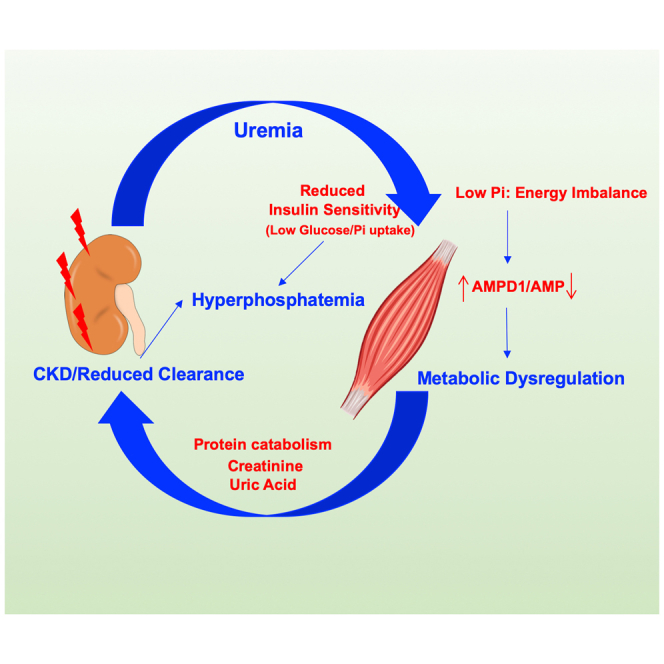

## Introduction

Sarcopenia (reduced skeletal muscle mass and strength) is extremely common in patients with chronic kidney disease (CKD), particularly in those with accompanying risk factors such as obesity, heart failure, diabetes, and aging.^1^ The consequences of sarcopenia are severe because it can shorten lifespan, cause frailty, increase the risk for falls, compromise the ability for individuals to care for themselves, and increase the need for wheelchair use.^2^^,^^3^^,^^4^^,^^5^^,^^6^ Thus, identifying the cause and treatment of sarcopenia remains an important goal.

Although sarcopenia was once considered the natural consequence of low physical activity and reduction in muscle mass that accompanies aging, sarcopenia is now recognized as a condition associated with low grade systemic inflammation, protein-energy wasting, altered muscle composition with fat infiltration and a catabolic state.^7^^,^^8^^,^^9^^,^^10^^,^^11^^,^^12^^,^^13^ Other associations include apoptosis, autophagy, mitochondria loss, intracellular activation of angiotensin II, overexpression of myostatin, and metabolic acidosis.^14^^,^^15^^,^^16^ This has led to the idea that sarcopenia might be part of a disease process that may require specific treatments.^17^^,^^18^^,^^19^^,^^20^

Muscle has a higher energy demand than most other organs. To facilitate it, phosphocreatine is stored so it can provide phosphate for ATP synthesis rapidly in times of need.^21^ However, both ATP and phosphocreatine depend on sufficient intracellular phosphate to be present, and if intracellular phosphate is chronically low, then ATP synthesis is markedly impaired. Indeed, intracellular phosphate depletion results in weakness and loss of muscle strength, with rhabdomyolysis, and even sarcopenia. Thus, we hypothesize that intracellular phosphate could represent the “Achilles’ heel” in muscle bioenergetics and possibly a key risk factor for sarcopenia.

Intracellular phosphate depletion is often associated with low serum phosphate and systemic phosphate depletion associated with poor intake or excessive loss of phosphate.^22^^,^^23^ However, intracellular phosphate depletion can also occur with normal or even high serum phosphate levels, especially in the setting of insulin resistance or deficiency.^24^ This is because insulin has an important role in the uptake of phosphate into cells through Na-phosphate transporters.^25^^,^^26^ In diabetic ketoacidosis, for example, subjects can present with marked hyperphosphatemia despite intracellular phosphate levels being low.^27^

In this regard, CKD is consistently associated with insulin resistance.^28^ Although serum phosphate is often high, there have been no studies that have investigated intracellular phosphate levels in the individual with CKD. However, studies suggest that phosphate stores are likely borderline or low, not only because of the insulin resistant state but also in part because dialysis removes not only extracellular but also intracellular phosphate.^29^ Indeed, a study of stable hemodialysis patients (that lacked sarcopenia or protein energy wasting) found that both intracellular phosphate and ATP levels fell during dialysis.^29^

Intracellular phosphate depletion is also associated with the activation of AMP deaminase (AMPD). AMPD is an AMP-dependent enzyme that converts AMP into inosine monophosphate (IMP) thus lowering nucleotide pools. Three main isoforms of AMPD have been described to date including the muscle-specific isoform AMPD1. As part of the purine degradation pathway, the last product of AMP metabolism via AMPD in humans is uric acid.^22^^,^^30^ Even though uric acid has antioxidant properties, high uric acid levels have been associated with inflammation, oxidative stress, and mitochondrial dysfunction^31^^,^^32^^,^^33^^,^^34^^,^^35^^,^^36^, and correlate with the severity of kidney disease^37^^,^^38^^,^^39^ and sarcopenia.^40^ Consistently, urate lowering therapies like the use of xanthine oxidase inhibitors reduce sarcopenia in dialysis patients.^41^

We hypothesized that one of the main consequences in CKD is that the reduced insulin sensitivity dysregulates phosphate homeostasis leading to intracellular phosphate depletion and AMPD1 activation, shifting muscle fibers into a catabolic-prone state.

## Results

### CKD-dependent sarcopenia is associated with activation of AMPD1 in the skeletal muscle

**Figure 1:**
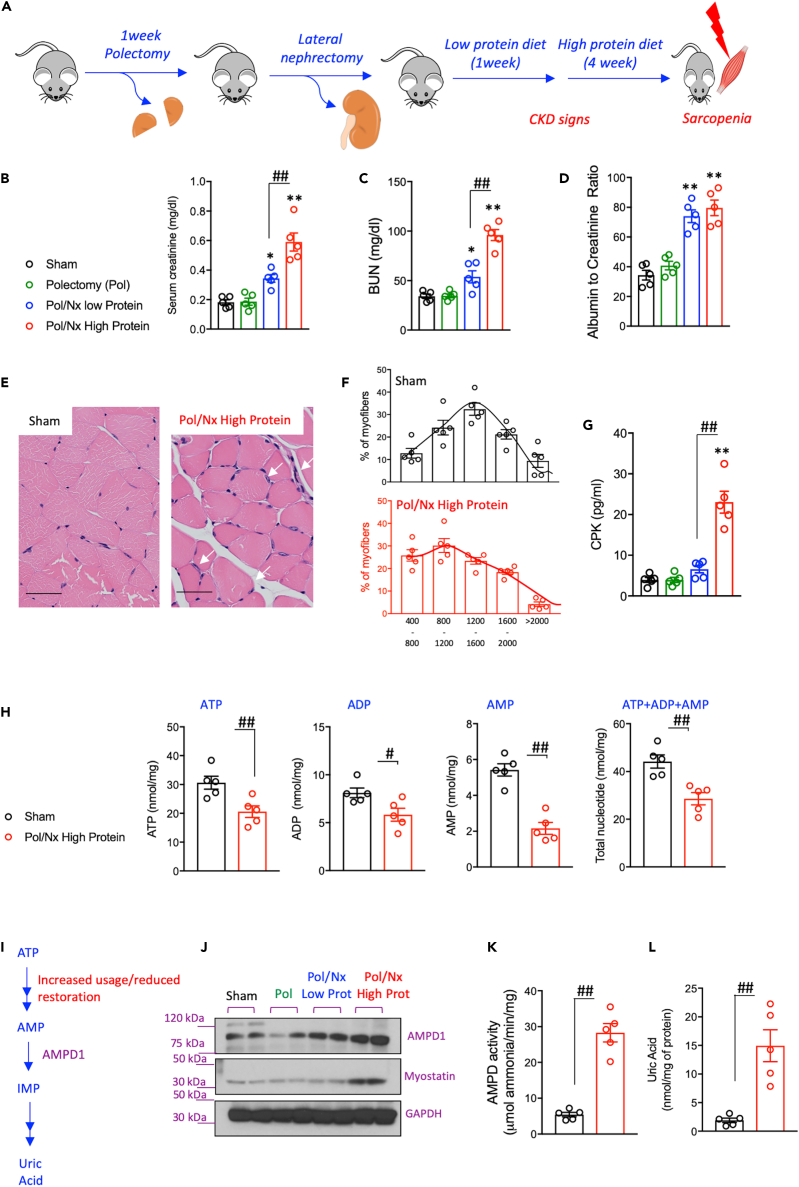
CKD-dependent muscle atrophy is associated with nucleotide turnover and AMPD1 activation (A) Schematic of the mouse model employed to induce CKD-dependent muscle atrophy. (B–D) Blood creatinine, urea and urinary albumin excretion in wild type mice undergoing sham operation (black) or at different states of the model: polectomy (green), lateral nephrectomy and low protein diet (green) and lateral nephrectomy and high protein diet (red). (E–G) (E) Representative H&E image of gastrocnemius of mice undergoing sham or with CKD. White arrows denote inflammatory cells. Bar = 25 μm (F) Cross-sectional analysis of myofiber sizes in sham and CKD mice showing a shift to the left (smaller size) (150 myofibers/muscle measured) (G) Plasma CPK levels in mice from same groups as in B). (H) Intramuscular nucleotide pool (ATP, ADP, AMP and total nucleotides) in sham and CKD mice. (I) Schematic of AMP metabolism via AMPD1 after ATP metabolism. (J) Representative western blot for AMPD1 and myostatin in gastrocnemius of mice from the same groups as in B). (K and L) Intramuscular AMPD activity and uric acid levels in sham and CKD mice. Statistical analysis: B-D and G) One way ANOVA followed by Tukey’s multiple comparison tests. H,K-L) two-tail t-test. ∗p <0.05 and ∗∗p <0.01 versus sham. #p <0.05 and ##p <0.01 n = 5 mice per group.

A model of progressive CKD modified from Zhang et al.^42^ was developed in mice. To this end, a two-stage surgery followed by a high protein diet for 4 weeks was performed as detailed in the methods section and in Figure 1A. The effect of this model on kidney function was assessed by measuring plasma creatinine and blood urea nitrogen (BUN) as well as the urine albumin to creatinine ratio. As shown in Figures 1B–1D, the addition of a high protein diet markedly caused renal dysfunction in mice. In parallel with worse kidney function, mice on a high-protein diet also developed sarcopenia compared to mice undergoing sham operation (Figures 1E–1G). Sarcopenia was denoted by a shift to the left in the size of muscle fibers (smaller fiber, Figures 1E and 1F) and a significant elevation in serum CPK levels (Figure 1G).

Analysis of the intramuscular nucleotide pool in mice with sarcopenia revealed a dramatic reduction in all intramuscular adenine nucleotides (40.9% reduction in ATP + ADP + AMP levels compared with sham operation and high protein diet, p< 0.01*,*Figure 1H). Remarkably, the major reduction is observed in AMP levels with over a 60% reduction between mice with sarcopenia and sham controls suggesting the presence of an active mechanism removing AMP from the muscle. In this regard, protein analysis from skeletal muscle extracts demonstrated the activation of AMPD1 (Figures 1I–1L) which participates in the deamination of AMP to inosine monophosphate as part of the purine degradation pathway, a catabolic route in which the final product in humans is uric acid (Figure 1I). Of interest, the activation of AMPD1 was associated with both a marked increase in its protein expression (Figure 1J) in parallel with increased myostatin, a well-described protein upregulated with muscle atrophy, and by a significant increase in overall activity (Figure 1K). As a result, intramuscular uric acid levels were significantly up-regulated in the muscle of sarcopenic mice (7.8-fold increase, p< 0.01).

### Reduced insulin-sensitivity in CKD drives intramuscular phosphate depletion in mice

**Figure 2:**
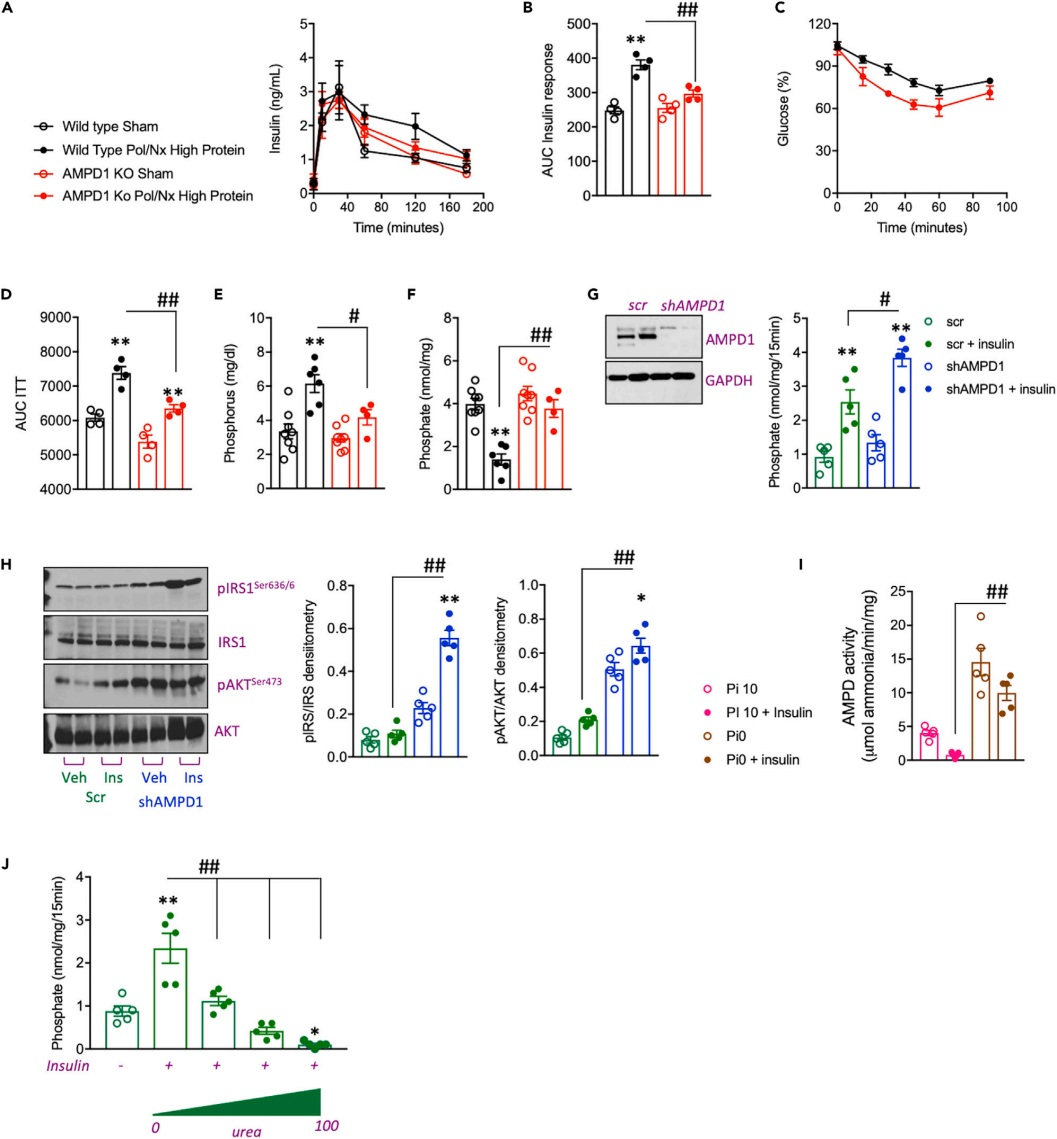
Reduced insulin sensitivity in CKD activates AMPD1 in the skeletal muscle (A and B) Plasma insulin levels over time (minutes) and area under the curve (AUC) in wild type (black) and AMPD1 KO (red) mice undergoing sham operation (open symbols) or after polectomy and lateral nephrectomy followed by 3 weeks of high protein diet (solid symbols) after an oral glucose (1.75 g/kg) tolerance test. (C and D) Plasma glucose levels and area under the curve (AUC) in wild type and AMPD1 KO mice undergoing CKD. (E) Plasma phosphate levels at sacrifice in the same mice as in A). (F) Intramuscular phosphate levels at sacrifice in the same mice as in A). (G) Representative western blot for AMPD1 and insulin dependent phosphate uptake in C2C12 myotubes control (scr, scramble) or silenced for AMPD1 (shAMPD1). (H) Representative western blot and densitometry for total and phosphorylated IRS1 and AKT in saline or insulin exposed control and AMPD1 deficient C2C12 myotubes. (I) AMPD activity in control and AMPD1 deficient C2C12 myotubes under 10 mM Pi (Pi10) or phosphate deprived (Pi0) conditions in the presence or absence of insulin. (J) Insulin-dependent phosphate uptake in control C2C12 myotubes in the presence of increasing amounts of urea. Statistical analysis: For B-D, F: One-way ANOVA followed by Tukey’s multiple comparison test. ∗∗p <0.01 versus respective strain sham. #p <0.05, ##p <0.01 n= 5 mice per group. For G, I-J, One-way ANOVA followed by Tukey’s multiple comparison test. ∗∗p <0.01 versus respective control. #p <0.05, ##p <0.01 Data graphed is the result of 5 independent replicates.

Insulin resistance is a common finding in patients with CKD, particularly in those requiring dialysis^43^^,^^44^^,^^45^^,^^46^ including in non-diabetic patients.^47^^,^^48^ Even though circulating glucose levels in 14-h fasting mice undergoing CKD-dependent sarcopenia or sham operation did not differ (116.2 ± 14.7 mg/dL in mice with sarcopenia versus 107.7 ± 18.7 mg/dL in sham), insulin levels following an oral glucose exposure were significantly higher in wild type mice with sarcopenia (Figures 2A and 2B, black symbols). Of interest, this increase seems to be the consequence of reduced clearance of insulin as there was not a significant difference in insulin peak release following glucose exposure (40′ after glucose challenge) between sarcopenia and sham. Rather, the marked difference in insulin levels corresponded to later time points (60′-180′ after glucose challenge) suggesting that the reduced clearance could be secondary to lower sensitivity of tissues to insulin. Notably, unlike wild type mice, similar insulin sensitivity was detected in mice deficient for AMPD1 (AMPD1 KO) undergoing sham operation or with CKD (Figures 2A and 2B, red symbols). This would suggest that the loss of AMPD1 in the skeletal muscle improves insulin sensitivity in CKD. Consistently, and as shown in Figures 2C and 2D, AMPD1 KO mice with CKD demonstrated better insulin sensitivity than wild type animals with the same degree of CKD as denoted by greater reduction in blood glucose levels following an intraperitoneal insulin injection (0.5 U/kg). Reduced insulin sensitivity was associated with greater plasma levels of phosphorus in wild type mice with CKD (6.15 ± 1.04 mg/dL versus 3.35 ± 1.23 mg/dL in sham, p< 0.01) than in AMPD1 KO animals (4.17 ± 0.88 mg/dL in AMPD1 KO mice with CKD versus 2.96 ± 0.68 mg/dL in sham, p< 0.01), Figure 2E. Furthermore, high plasma phosphate in wild type but not in AMPD1 KO mice with CKD correlated with a significant reduction in intramuscular phosphate (1.40 ± 0.45 nmol/mg in mice with CKD versus 3.98 ± 0.74 nmol/mg in sham, p< 0.01, Figure 2F).

### Urea impairs insulin signaling and phosphate uptake driving AMPD1 activity in human myotubes

To better characterize the consequence of reduced insulin sensitivity in CKD and whether it is associated with AMPD1 activation in the skeletal muscle, we evaluated the response to insulin of murine C2C12 myotubes. We first assessed insulin signaling in control and AMPD1 knockout myotubes. Exposure of myotubes to insulin (25 μM) resulted in a significant increase in sodium-dependent phosphate transport as previously described in other cell types,^25^^,^^26^^,^^49^^,^^50^Figure 2G. Importantly, and as shown in Figure 2G, phosphate transport rates were up-regulated in AMPD1 deficient myotubes compared to control cells (2.54 ± 0.78 nmol/mg/15′ in control myotubes versus 3.84 ± 0.56 nmol/mg/15′ in AMPD1 deficient myotubes, p< 0.05, Figure 2G) in parallel with a substantially higher activation of insulin-dependent signaling targets including insulin-receptor 1 (IRS1) and AKT as denoted by analysis of their phosphorylated state by western blot (Figure 2H).

To test whether insulin-dependent intracellular phosphate levels could modify AMPD activity, we then analyzed AMPD activity in myotubes exposed to control medium containing 10 mM phosphate or to phosphate deprived medium in the presence or absence of insulin. Addition of insulin (25 μM) reduced AMPD activity in control cells (Figure 2I). AMPD activity was significantly higher in phosphate deprived cells compared to control (4.04 ± 0.86 μmol ammonia/min/mg in control myotubes versus 14.55 ± 4.53 μmol ammonia/min/mg in phosphate deprived myotubes, p< 0.01, Figure 2I). Of interest, insulin failed to lower AMPD activity in phosphate deprived cells thus indicating that the mechanism whereby insulin regulates AMPD activity is mediated by intracellular phosphate. Furthermore, and to determine how CKD reduced the sensitivity of the skeletal muscle to insulin, we analyzed insulin-dependent phosphate transport in the presence of urea which is commonly accumulated in the blood of patients with CKD. As shown in Figure 2J, urea significantly impaired insulin-dependent phosphate transport in a dose-dependent manner (from 0 to 100 mg/dL) indicating that the high blood urea nitrogen (BUN) present in patients with CKD may be responsible for the reduced insulin sensitivity in these subjects.

### AMPD1 activation in the skeletal muscle contributes to the progression of CKD in mice

**Figure 3:**
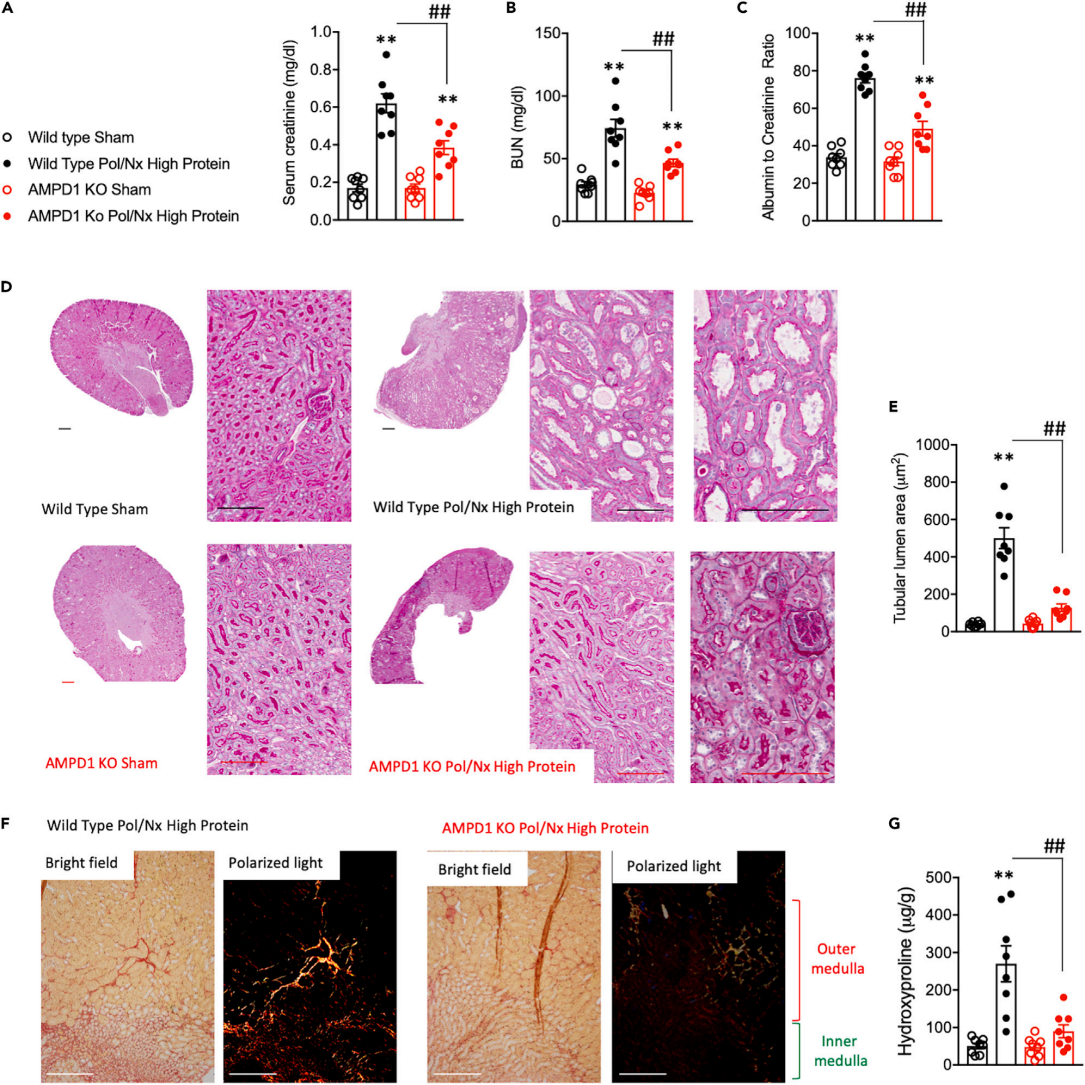
Deletion of AMPD1 ameliorates CKD in mice (A–C) Plasma creatinine, plasma urea and urinary albumin excretion in wild type (black) and AMPD1 KO (red) mice undergoing sham operation (open symbols) or after polectomy and lateral nephrectomy followed by 4 weeks of high protein diet (solid symbols): (D) Representative PAS kidney images from wild type or AMPD1 KO mice undergoing sham or with CKD. Bar = 50 μm (E) Tubular lumen area (dilatation) in the same mice as in A) (50–100 tubules/kidney measured) (F) Representative picrosirius red staining under brightfield and polarized light in renal outer and inner medulla of wild type or AMPD1 KO mice undergoing CKD. Bar = 50 μm (G) Renal hydroxyproline levels in the same mouse groups as in (A). Statistical analysis: One-way ANOVA followed by Tukey’s multiple comparison test. ∗∗p <0.01 versus respective strain sham. ##p <0.01 n = 8 mice per group.

To test the importance of muscle AMPD1 activation in CKD, we carried out the same approach depicted in Figure 1A in wild type control and AMPD1 KO mice. Of interest, at the end of the 4 weeks exposure to the high protein diet, AMPD1 KO mice demonstrated significantly better renal function than wild type counterparts as denoted by reduced plasma levels of creatinine (0.62 ± 0.14 mg/dL in wild type versus 0.38 ± 0.10 in AMPD1 KO, p< 0.01, Figure 3A), BUN (74.38 ± 20.11 mg/dL in wild type versus 46.83 ± 8.46 in AMPD1 KO, p< 0.01, Figure 3B) and urinary albumin excretion (76.13 ± 7.25 urinary albumin to creatinine ratio in wild type versus 49.13 ± 11.17 in AMPD1 KO, p< 0.01, Figure 3C). Consistent with improved renal function, kidney injury was reduced in AMPD1 deficient mice (Figures 3D–3G), particularly in the cortical and outer medullary S1 and S2 segments of the proximal tubule in which tubules from AMPD1 KO demonstrated a better integrity of the brush border area (Figure 3D) and reduced tubular dilatation (Figure 3E). Similarly, fibrosis, a hallmark of CKD progression, was substantially reduced in AMPD1 KO mice as denoted by less collagen deposition particularly in outer and inner medullary strips (Figure 3F) and significantly lower levels of hydroxyproline, a marker of severity in fibrotic tissues (270.1 ± 136.5 μg/g in wild type versus 90.1 ± 49.1 in AMPD1 KO, p< 0.01, Figure 3G). Importantly, the beneficial effects observed in AMPD1 KO mice undergoing CKD were not a consequence of lower intake of the high protein diet as no significant differences were observed in daily (2.62 ± 0.22 g/day versus 2.34 ± 0.16 in wild type mice with CKD) and 4-week cumulative food consumption between strains.

### AMPD1 blockade protects against sarcopenia independently of the severity of the renal dysfunction

**Figure 4:**
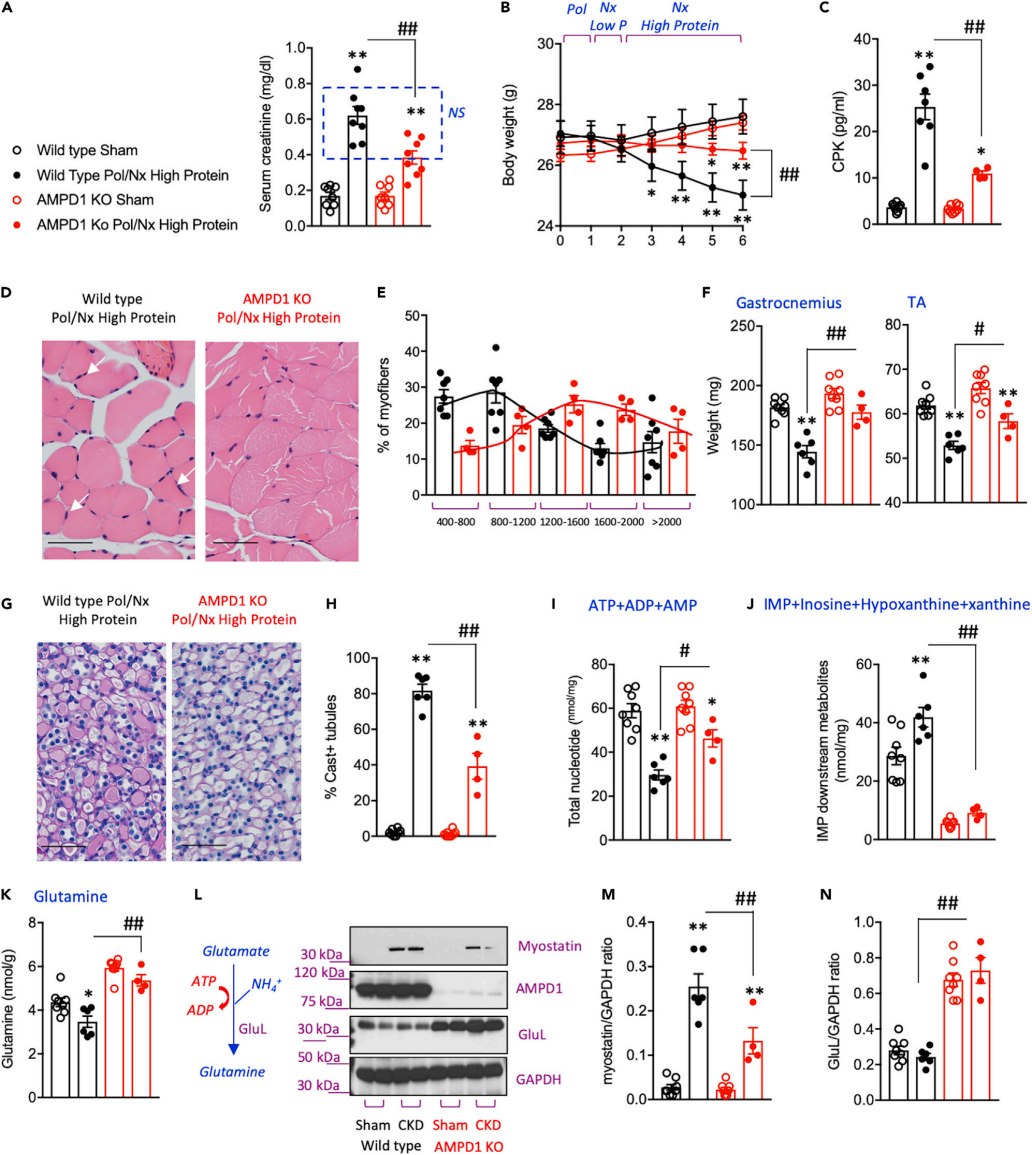
Deletion of AMPD1 ameliorates muscle atrophy in CKD-matched mice (A) Plasma creatinine in wild type (black) and AMPD1 KO (red) mice undergoing sham operation (open symbols) or after polectomy and lateral nephrectomy followed by 4 weeks of high-protein diet (solid symbols). Creatinine values of CKD-matched mice chosen for further sub-analysis (6 wild type and 4 AMPD1 KO) are included in the blue square. (B) Body weight change during the length of the study in the same groups as in (A) (C) Plasma CPK levels in mice from same groups as in (A). (D) Representative H&E image of gastrocnemius of CKD-matched wild type and AMPD1 KO mice. White arrows denote inflammatory cells. Bar = 25 μm (E) Cross-sectional analysis of myofiber sizes in CKD-matched wild type and AMPD1 KO mice (150 myofibers/muscle measured) (F) Muscle weight of the same mice as in C). (G) Representative PAS staining of kidney medullas demonstrating greater tubular cast formation in wild type mice undergoing CKD compared to AMPD1 KO. Bar = 50 μm. (H) Quantitative analyses of cast positive tubules in CKD-matched wild type and AMPD1 KO mice (75–125 tubules/kidney measured) (I) Intramuscular nucleotide pool in the same mice as in (A). (J) Intramuscular AMPD-dependent downstream AMP-metabolites in the same mice as in (A). (K) Intramuscular glutamine levels in the same mice as in (A). (L–N) Representative western blot and densitometry analysis for myostatin, AMPD1 and glutamine synthetase (GluL) in gastrocnemius of mice from the same groups as in A). Statistical analysis: One-way ANOVA followed by Tukey’s multiple comparison test. ∗p <0.05 and ∗∗p <0.01 versus respective strain sham. #p <0.05, ##p <0.01 n = 4–8 mice per group.

The data presented in Figure 3 would suggest that any potential benefit in blocking AMPD1 on sarcopenia could be the consequence of just improved renal function. Therefore, because the improvement in kidney function could potentially modify the risk for sarcopenia, we performed a subanalysis in which we grouped wild type and AMPD1 KO mice with similar creatinine (0.58 ± 0.12 mg/dL in wild type n = 7 mice versus 0.49 ± 0.07 in AMPD1 KO mice n = 4, *P* = not significant, Figure 4A) and BUN values (65.33 ± 11.38 mg/dL in wild type n = 7 mice versus 52.50 ± 7.68 in AMPD1 KO mice n = 4, *P* = not significant). During the 4 weeks on a high protein diet, weight loss in AMPD1 KO mice was not as marked as in wild type animals with similar kidney function (Figure 4B) with lower levels of creatine phosphokinase (CPK) in plasma (Figure 4C) indicative of improved energy balance and reduced muscle catabolism associated with the blockade of AMPD1. No significant differences in caloric intake were found between wild type and AMPD1 KO in the mice included in this sub-analysis. Consistently, AMPD1 KO mice with CKD demonstrated reduced muscle inflammation (Figure 4D) and preserved myofiber size (Figure 4E). As a result, the weights of gastrocnemius and tibialis anterior (TA) were significantly greater in AMPD1 KO mice compared to wild type animals with similar kidney function (Figure 4F) and muscle-derived casts in renal tubules were substantially reduced in AMPD1 KO mice (Figures 4G and 4H). Analysis of nucleotide pools revealed that the expected improvement in total nucleotide levels in AMPD1 KO mice (Figure 4I) was characterized by a much higher concentration of free AMP (2.41-fold increase in AMPD1 KO mice versus wild type, p< 0.01), ATP (0.32-fold increase in AMPD1 KO mice versus wild type, p< 0.05), and the AMP to ATP ratio (0.33 ± 0.11 AMP/ATP ratio in wild type versus 0.59 ± 0.11 AMP/ATP ratio in AMPD1 KO, p = 0.014). Of interest, the significant increase in AMP levels in the muscle of AMPD1 KO mice is paralleled with reduced clearance via AMPD as levels of intramuscular IMP and its downstream metabolites inosine, hypoxanthine and xanthine; are markedly low in AMPD1 KO mice (Figure 4J). Metabolic dysregulation secondary to reduced insulin sensitivity and negative energy balance in the skeletal muscle of mice with CKD leads to the utilization of protein as energy fuel and therefore to a switch favoring protein catabolism over synthesis. In this regard, glutamine is one of the most important amino acids primarily produced by the skeletal muscle whose supplementation has been shown to prevent loss of muscle mass.^51^^,^^52^^,^^53^^,^^54^ Intramuscular glutamine levels in AMPD1 KO mice with CKD are significantly higher than wild type animals (Figure 4K) in parallel with an up-regulated expression of glutamine synthetase (GluL), the enzyme that produces glutamine from glutamate and ammonia (Figures 4L–4N). Furthermore, we found that GLuL up-regulation in AMPD1 KO mice was associated with a reduction in intramuscular levels of the anti-myogenic factor, myostain, suggesting an inverse correlation between these two enzymes in the regulation of muscle mass.

## Discussion

**Figure 5:**
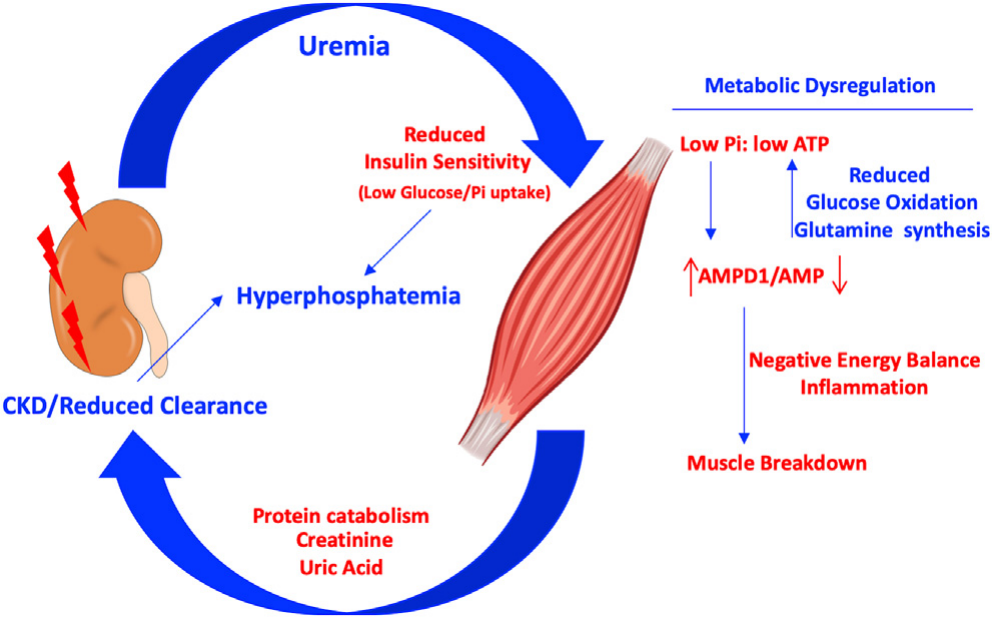
Potential mechanism or kidney-muscle interplay in the progression of CKD and sarcopenia In CKD, reduced clearance elevates plasma levels of urea. In turn, increased uremia impairs insulin action on the skeletal muscle leading to a reduction of both insulin-dependent glucose and phosphate uptake causing metabolic dysregulation. Hyperphosphatemia is then the consequence of both reduced renal clearance and low uptake by the skeletal muscle. Metabolic dysregulation in the skeletal muscle is characterized by low phosphate, reduced glucose oxidation and protein synthesis and overall low ATP and phosphocreatine levels with AMPD1 activation. Hyperactive AMPD1, in turn, decreases the amount of AMP while promoting the formation of uric acid in the purine degradation pathway. As a result of a negative energy balance in the muscle, inflammation and protein turnover is manifest thus leading to muscle atrophy. In consequence, muscle-derived products from protein catabolism and AMPD1 activation are released to the circulation to further contribute to the progression of CKD.

Sarcopenia or muscle atrophy is a common consequence in CKD but the mechanism whereby declined renal function reduces muscle mass is still unclear. The data from our study supports a novel model of CKD-dependent muscle atrophy as depicted in Figure 5which is mediated by the activation of AMPD1 in the skeletal muscle and the removal of free AMP. We propose that the activation of AMPD1 as the result of low intramuscular phosphate is the consequence of a marked reduced sensitivity of myofibers to insulin. In this regard, insulin resistance is commonly found in patients with CKD^55^^,^^56^^,^^57^^,^^58^^,^^59^^,^^60^ in which the skeletal muscle represents the primary site of reduced insulin sensitivity. The proposed etiology of reduced insulin sensitivity in CKD is multifactorial and include physical inactivity, protein energy wasting, chronic inflammation,^61^ vitamin D deficiency,^62^ metabolic acidosis^63^, urea retention^64^, and renal anemia^65^^,^^66^ among others. In the present study, we found some of these factors taking place in mice undergoing CKD and sarcopenia including lower caloric intake, inflammation and high uremia. The effect of high urea on insulin resistance is mediated by a decrease in erythropoietin production by uremic toxins causing anemia. However, here we demonstrate that uremia decreases insulin sensitivity dose dependently in isolated myotubes suggestive of a local effect in the skeletal muscle independently of anemia. This is consistent with the findings of D’Apolito et al.^67^ suggesting that urea-dependent generation of reactive oxygen species and oxidative stress is the cause of muscular insulin resistance in mice with kidney dysfunction. This supports previous studies suggesting that improving insulin sensitivity could be beneficial in sarcopenia and the progression of CKD.^68^^,^^69^

Even though insulin sensitivity is traditionally associated with glucose transport and utilization, insulin plays an important role in promoting phosphate uptake as well.^25^^,^^26^ Insulin dependent phosphate uptake is sodium dependent and mediated by the slc20 family of transporters. Insulin-dependent phosphate uptake is particularly relevant in the skeletal muscle as phosphate is necessary for energy storage as ATP and phospho-creatine necessary for glucose phosphorylation and glycolysis. Therefore, a low intramuscular phosphate state would aggravate the low insulin sensitivity already present in CKD. Consistently, the loss of phosphate transport in slc20a1/Pit1 and slc20a2/Pit2 deficient mice causes myofiber dysfunction and atrophy.^70^ It is important to note that low intramuscular phosphate levels contrasts with circulating hyperphosphatemia commonly found in subjects with CKD. Therefore, the high phosphate in CKD would be the consequence of both decreased renal function and lower insulin-dependent phosphate uptake. Current strategies to regulate phosphate in CKD are limited to control its plasma levels. However, our data indicate that in CKD there is also a significant metabolic phosphate dysregulation intracellularly particularly in the skeletal muscle which needs to be repaired. Consistently, a recent NMR study of dialysis patients has confirmed that dialysis lower intracellular phosphate and ATP levels.^29^ This documents the insufficient energy backup in the muscle of dialysis patients bringing attention that simply removing extracellular phosphate may not fully address the problems in phosphate metabolism in CKD. Therefore, strategies aimed to reduce plasma phosphate like the use of phosphate binders or reducing intake should be better adjusted or given in combination with insulin sensitizing drugs particularly to those CKD subjects at greater risk of developing sarcopenia. In accordance, a recent meta-analysis of 16,800 type 2 diabetic patients^71^ reported that metformin, an insulin sensitizing drug, was associated with reduced risk of sarcopenia in patients with type 2 diabetes. It is also notable that exercise training programs, an established method to improve insulin resistance, improve both phosphate removal and reduce the risk of sarcopenia in dialysis patients.^72^^,^^73^

Phosphate regulates multiple molecular and signaling pathways. Of these, phosphate and phosphoinositides are known to inhibit AMPD activity *in vivo* and *in vitro*^74^ and low intracellular phosphate leads to AMPD activation.^75^ In this regard, we have previously shown that the activation of another isoform of AMPD, AMPD2, in insulin-insensitive and phosphate-deprived hepatocytes promoted endogenous glucose production from gluconeogenic factors in response to starvation.^75^ This would suggest that the effects of AMPD activation via low insulin sensitivity and low intracellular phosphate in CKD may affect multiple organs. Therefore, further studies aimed to analyze the systemic effects of AMPD activation in CKD are warranted.

Finally, one of the more relevant findings of our study is the spontaneous up-regulation and activation of glutamine synthetase (GluL) and the high levels of glutamine observed in the skeletal muscle of AMPD1 KO mice. Glutamine is one of the most abundant essential aminoacids and fuel source of several cell types (enterocytes and immune cells), a precursor of purine and pyrimidine synthesis and a negative regulator of protein catabolism. Even though it is still unclear whether glutamine supplementation prevents sarcopenia, beneficial effects of glutamine on muscle atrophy and muscle wasting have been reported.^51^^,^^54^^,^^76^ Importantly, our data support another potential benefit of glutamine synthesis in CKD besides the production of glutamine. Synthesis from glutamate requires ammonia as the nitrogen donor and therefore, the higher metabolic rate through this pathway in AMPD1 KO mice would help both detoxify ammonia and reduce urea production to ameliorate kidney dysfunction and improve insulin sensitivity. Thus, reduced ammonia and urea production via glutamine synthesis may therefore explain in part why AMPD1 deficient mice demonstrate improved renal function in our model compared to wild type counterparts.

In conclusion, we suggest a role for insulin resistance, intracellular phosphate depletion, and activation of AMP deaminase in the pathogenesis of sarcopenia in CKD. Measures that increase intracellular phosphate and improve insulin sensitivity and restore energy pools may represent a new approach for preventing and reversing sarcopenia.

### Limitations of the study

Besides AMPD1, AMPD3 is also expressed in the skeletal muscle. In this regard, some studies indicate that the expression of AMPD1 and AMPD3 genes may be coordinated in myocytes to effect production of an AMPD holoenzyme.^77^^,^^78^ Therefore, both isoforms may be required for proper AMPD activity. In this regard, Miller et al.^79^ demonstrated that the intramuscular overexpression of AMPD3 in mice and C2C12 myotubes depleted AMP levels and caused muscle atrophy and suggest the importance of AMPD3 in muscle waste and sarcopenia. However, it is important to note that our results indicate that the depletion of AMPD1 reduces IMP and its downstream metabolites in the muscle by >80% in both sham and CKD mice, indicating that AMPD1 is the main contributor of AMP deamination in the skeletal muscle. This is consistent with previous reports^80^ in which AMPD1 deficiency led to non-detectable IMP levels in the muscle of mice at baseline, following an exercise protocol or undergoing local ischemia Similarly, It would be helpful to know if the improvement in muscle mass was associated with an improvement in muscle strength. Ideally, it would great to knockout AMPD1 after kidney disease was induced to assure equivalent kidney disease in both groups. The studies were also performed in mice, which does not guarantee similar findings in humans.

## STAR★Methods

### Key resources table

**Table:** 

REAGENT or RESOURCE	SOURCE	IDENTIFIER
**Antibodies**		

Anti-mouse Myostatin	Proteintech	19142-1-AP; RRID:AB_10638615
Anti-mouse AMPD1	Proteintech	19780-1-AP; RRID:AB_10644281
Anti-mouse GLuL	Cell Signaling	80636; RRID:AB_2799956
Anti-mouse pIRS1^Ser636/639^	Cell Signaling	2388; RRID:AB_330339
Anti-mouse IRS1	Cell Signaling	2382; RRID:AB_330333
Anti-mouse pAKT^Ser473^	Cell Signaling	4058; RRID:AB_331168
Anti-mouse AKT	Cell Signaling	9272; RRID:AB_32982
Anti-mouse GAPDH	Cell Signaling	5174; RRID:AB_10622025
Goat Anti-Rabbit IgG HRP conjugated	Cell Signaling	7074; RRID:AB_2099233
Goat Anti-Mouse IgG HRP conjugated	Cell Signaling	7076; RRID:AB_330924

**Chemicals, peptides and recombinant proteins**		

Glucose	Sigma	G8270
Insulin (human)	Sigma	91077C
Urea	Sigma	U5378
DMEM, high glucose	Sigma	11965
DMEM, high glucose, no phosphates	Sigma	11971
Hematoxyline	Thermo Scientific	22-110-639
Eosin-Y	Fisher Scientific	SE23-500D
Magnesium Chloride	Sigma	M8266
Sodium Vanadate	Sigma	590088
Triton-X	Millipore	MTX15681
Tween 20	MP Biomedicals	MP1TWEEN201
Puromycin dihydrochloride	Santa Cruz Biotechnologies	sc-108071

**Critical commercial assays**		

Phosphate Determination Kit	Biovision	K410
Glutamate Determination Kit	Biovision	K629
Glucose Determination Kit	Biovision	K606
Creatinine Determination Kit	Pointe Scientific	C7548
Uric Acid Determination Kit	Bioassay Systems	DIUA-250
Creatine Phosphokinase Kit	Biovision	K477
Blood Urea Nitrogen Determination Kit	Bioassay Systems	DIUR-100
Albumin Determination Kit	Ethos Biosciences	Albuwell M
Anti-Mouse Insulin ELISA	Crystal Chem	90080

**Deposited data**		

Original Western blot and histology images	Mendeley Data	https://data.mendeley.com/datasets/zjwhhpzgvm/1

**Oligonucleotides**		

shRNA against human AMPD1	Santa Cruz Biotechnologies	Sc-141059-v

**Other**		

Glucometer	Accu-Check	Accu-Check Guide Meter
Glucose Strips	Accu-Check	Accu-Check Guide Test Strips
C2C12 cell line	ATCC	CRL-1772 RRID:CVCL_0188

### Resource availability

#### Lead contact

Further information and requests for resources and reagents should be directed and will be fulfilled by the Lead Contact, Miguel A. Lanaspa (Miguel.lanaspagarcia@cuanschutz.edu).

#### Materials availability

Mouse lines generated in this study are available for any researcher upon reasonable request.

### Experimental model and subjectdetails

#### Study approval

All animal experiments were conducted with adherence to the NIH Guide for the Care and Use of Laboratory Animals.^81^ All animal experiments and procedures were approved by the Animal Care and Use Committee of the Veterans Affairs Medical Center and the University of Colorado (Aurora, CO).

#### Animals

CKD-induced sarcopenia was carried out as in^42^ with modifications. Male 10–12 weekold AMPD1 KO (*B6;129_Ampd1*^*tm1b(KOMP)Wtsi*^) or wild type littermates underwent subtotal nephrectomy in 2 stages. In the first stage, ∼70% of the right kidney was removed. Seven days later, the left kidney was removed, and mice fed a 12% protein diet (Envigo, Indianapolis, IN, USA) to minimize mortality from uremia. One week later, mice were fed a 40% protein diet for 4 weeks (TD_90018, Envigo, Indianapolis, IN, USA) to induce advanced CKD. Sham-treated control mice underwent both surgeries without damaging the kidneys and were fed the same diets. All mice were housed with 12-h light-dark cycles. Body weights and food consumption were assessed daily.

### Method details

#### Biochemical analysis

Blood was collected in Microtainer tubes (BD) from cardiac puncture of mice under isoflurane, and serum was obtained after centrifugation at 13,000 rpm for 2 minat room temperature. Serum parameters were determined biochemically with commercially available kits following the manufacturer’s instructions Phosphate: K410, Creatine phosphokinase (CPK): K777, Glutamate: K629 and Glucose K606 from Biovision, Milpitas, CA; Creatinine: C7548, Pointe Scientific, Canton, MI; Blood urea nitrogen (BUN): DIUR-100 and Uric Acid: DIUA-250 Bioassay Systems, Hayward, CA; Albumin: Albuwell M (Ethos Biosciences, Logan Township, NJ); Insulin: Ultra-sensitive mouse insulin ELISA kit, 90,080, Crystal Chem, Elk Grove Village, IL.

#### Histopathology

Formalin-fixed paraffin-embedded kidney and muscle sections were stained with periodic acid-Schiff (PAS). Histological examination was performed across the entire cross section of the kidney and muscle from each mouse. The distribution of myofiber sizes was calculated on the basis of analysis of 750 myofibers per mouse (approximately 150 fibers/section). Tubular lumen area and cast formation were measured with an Aperio Scanscope. Kidneys were stained with Picro Sirius Red to assess for fibrosis as previously described.^82^ Images were captured on an Olympus BX51 microscope equipped with a 4-megapixel Macrofire digital camera (Optronics, Goleta, CA) using the PictureFrame Application 2.3 (Optronics). Composite images were assembled with the use of Adobe Photoshop. All images in each composite were handled identically.

#### Nucleotide determination

Tissues were collected and snap frozen in liquid nitrogen before being homogenized in ice-cold in a buffer containing perchloric acid (0.5N) and EDTA (5 mM). Homogenates were then centrifuged, and supernatants neutralized with ice-cold 1 N KOH. Homogenates were separated UPLC and concentrations of total adenine nucleotides and degradation products quantified at 215 nm. Metabolite data was normalized to protein data obtained from resuspended pellets after neutralization.

#### Oral glucose and insulin tolerance tests

For determination of glucose-dependent insulin levels, mice were fasted for 8 h. At baseline, blood was obtained from a tail snip and glucose measured using a One-touch Ultra 2 glucometer. The remaining blood was processed for plasma that was later used to determine the fasting insulin levels. The mice then received 1.75 g/kg body weight of a glucose solution (Sigma, G8769) in tap water by oral gavage. After the administration of glucose, dried blood and tissue were quickly removed from the tail wound and blood was collected again to prepare the plasma samples for measuring insulin levels. All of the plasma samples were frozen after collection and assayed later by ELISA (Mouse/Rat insulin kit, 90,080, Crystal Chem) according to the manufacturer’s protocol. For insulin tolerance test, mice were fasted for 4 h and blood collected for glucose determination as described above. Insulin (0.5 U/kg) was administered intraperitoneally with a 27G needle.

#### Western blot analysis

Protein lysates were prepared from mouse tissue or C2C12 cells using lysis buffer containing 0.3% Triton X-. Protein content was determined by the BCA protein assay (Pierce, Rockford, IL). Total protein (50 μg) was separated by SDS-PAGE [10% (w/v)] and transferred to PVDF membranes (Bio-Rad, Hercules, CA). Membranes were first blocked for 1 hat 25°C in 4% (w/v) instant milk dissolved in 0.1% Tris-buffered saline with Tween 20 TBS (TTBS) and incubated with the following primary rabbit-raised antibodies (1:1,000 dilution in TTBS): Myostatin (19142-1-AP, Proteintech; RRID:AB_10638615), AMPD1 (19780-1-AP; RRID:AB_10644281), GLUL (80,636, Cell Signaling; RRID:AB_2799956), pIRS1^Ser636/639^ (2388, Cell Signaling; RRID:AB_330339), IRS1 (2382, Cell Signaling; RRID:AB_330333), pAKT^Ser473^ (4058 Cell Signaling; RRID:AB_331168), AKT (9272, Cell signaling; RRID:AB_32982) and GAPDH (5174, Cell Signaling; RRID:AB_10622025) and visualized using an anti-rabbit (no. 7074) horseradish peroxidase (HRP)-conjugated secondary antibody (1:2,000, Cell Signaling) using the HRP Immunstar detection kit (Bio-Rad). Chemiluminescence was recorded with an Image Station 440CF, and results were analyzed with the 1D Image software (Kodak Digital Science, Rochester, NY). Data for proteins of interest are expressed normalized to GAPDH expression.

#### Studies in C2C12 myotubes

C2C12 cells were obtained from the AmericanType Cell Culture (ATCC, CRL-1772; RRID:CVCL_0188), grown and differentiated as per the ATCC recommendations. Stable deletion of AMPD1 was carried out with lentiviral particles containing shRNAs against murine AMPD1 (sc-141052-v, Santa Cruz Biotechnologies) followed by clonal selection with puromycin and AMPD1 expression analysis by western blot. Differentiated C2C12 myotubes were exposed to different compounds including insulin (91077C, Sigma), urea (U5378, Sigma) and complete 10 mM phosphate (11,965, Gibco) or phosphate free (11,971, Gibco) medium. Phosphate uptake assays were performed with [^32^P]orthophosphoric acid (120 μM) as radiotracer under constant sodium concentration (120 mM). Transport values were normalized by calculated (BCA, pierce) protein concentration.

### Quantification and statistical analysis

#### Statistical analysis

All numerical data are presented as the mean ± s.e.m. Independent replicates for each data point (n) are identified in figure legends. Data graphics and statistical analysis were performed using Prism 5 (GraphPad). Data without indications were analyzed by one-way ANOVA, Tukey post hoc test. A value of P < 0.05 was regarded as statistically significant. Animals were randomly allocated in each group using randomizer (www.randomizer.org). Power calculations for the number of animals assigned to each group were based on our previous publications and designed to observe a greater than 15% difference in body weight difference between groups. In general, an *n* of 7-8 mice per group was used. No animals were excluded from the study and whenever possible experiments were done in a blond fashion. For example, for data analysis, except for western blot, single samples (plasma, homogenates,…) were first codified and decoded after determination. Similarly, histological records and scoring were done in a blind fashion.

## Data Availability

• This study did not generate unique datasets or code. • This study did not generate new unique reagents, cell lines, or mouse lines. • Original western blot and histology images have been deposited at Mendeley and are publicly available as of the date of publication. The DOI is listed in the key resources table.
